# Non-cross Bridge Viscoelastic Elements Contribute to Muscle Force and Work During Stretch-Shortening Cycles: Evidence From Whole Muscles and Permeabilized Fibers

**DOI:** 10.3389/fphys.2021.648019

**Published:** 2021-03-29

**Authors:** Anthony L. Hessel, Jenna A. Monroy, Kiisa C. Nishikawa

**Affiliations:** ^1^ Institute of Physiology II, University of Muenster, Muenster, Germany; ^2^ W.M. Keck Science Department, Claremont Colleges, Claremont, CA, United States; ^3^ Department of Biological Sciences, Northern Arizona University, Flagstaff, AZ, United States

**Keywords:** 2,3-butanedione monoxime, calcium-dependence, doublet potentiation, skeletal muscle, titin, work loop, history-dependence

## Abstract

The sliding filament–swinging cross bridge theory of skeletal muscle contraction provides a reasonable description of muscle properties during isometric contractions at or near maximum isometric force. However, it fails to predict muscle force during dynamic length changes, implying that the model is not complete. Mounting evidence suggests that, along with cross bridges, a Ca^2+^-sensitive viscoelastic element, likely the titin protein, contributes to muscle force and work. The purpose of this study was to develop a multi-level approach deploying stretch-shortening cycles (SSCs) to test the hypothesis that, along with cross bridges, Ca^2+^-sensitive viscoelastic elements in sarcomeres contribute to force and work. Using whole soleus muscles from wild type and *mdm* mice, which carry a small deletion in the N2A region of titin, we measured the activation- and phase-dependence of enhanced force and work during SSCs with and without doublet stimuli. In wild type muscles, a doublet stimulus led to an increase in peak force and work per cycle, with the largest effects occurring for stimulation during the lengthening phase of SSCs. In contrast, *mdm* muscles showed neither doublet potentiation features, nor phase-dependence of activation. To further distinguish the contributions of cross bridge and non-cross bridge elements, we performed SSCs on permeabilized psoas fiber bundles activated to different levels using either [Ca^2+^] or [Ca^2+^] plus the myosin inhibitor 2,3-butanedione monoxime (BDM). Across activation levels ranging from 15 to 100% of maximum isometric force, peak force, and work per cycle were enhanced for fibers in [Ca^2+^] plus BDM compared to [Ca^2+^] alone at a corresponding activation level, suggesting a contribution from Ca^2+^-sensitive, non-cross bridge, viscoelastic elements. Taken together, our results suggest that a tunable viscoelastic element such as titin contributes to: (1) persistence of force at low [Ca^2+^] in doublet potentiation; (2) phase- and length-dependence of doublet potentiation observed in wild type muscles and the absence of these effects in *mdm* muscles; and (3) increased peak force and work per cycle in SSCs. We conclude that non-cross bridge viscoelastic elements, likely titin, contribute substantially to muscle force and work, as well as the phase-dependence of these quantities, during dynamic length changes.

## Introduction

As scientific theories evolve, paradigms often shift as seemingly obscure facts that resisted explanation by an accepted theory take on new importance (Kuhn, 1962). In the sliding-filament, swinging cross bridge theory of muscle contraction (Huxley and Hanson, 1954; Huxley and Niedergerke, 1954; Huxley, 1957), the isometric force-length relationship (Gordon et al., 1966), and the isotonic force-velocity relationship (Huxley, 1957) played important roles in establishing the theory as fact. However, several key observations including enhancement of force with stretch and depression of force with shortening resisted explanation by the accepted theory (Abbott and Aubert, 1952; Edman, 1975; Edman et al., 1982; Tsuchiya and Sugi, 1988). The perceived importance of these history-dependent muscle properties has increased over recent decades with the recognition that they play essential roles in human and animal movement by enhancing work and power output, dissipating force, and providing instantaneous stabilization during unexpected perturbations (Daley and Biewener, 2011; Nishikawa et al., 2013; Seiberl et al., 2013). Additionally, there is increasing recognition that muscle models based on the isometric force-length and isotonic force-velocity relationships fail to predict muscle force during dynamic *in vivo* movement (Siebert et al., 2008; Lee et al., 2013; Dick et al., 2017), perhaps because a critical element is missing (Rode et al., 2009; Heidlauf et al., 2017; Nishikawa et al., 2018; Nishikawa, 2020).

Accumulating evidence suggests that Ca^2+^-sensitive, non-cross bridge, viscoelastic elements in sarcomeres of skeletal muscles (Monroy et al., 2012; Colombini et al., 2016; Nishikawa et al., 2019), are responsible for history-dependent muscle properties during changes in length (Nocella et al., 2014; Nishikawa, 2016; Herzog, 2018). It has also become increasingly accepted that the giant titin protein is a Ca^2+^-sensitive, viscoelastic element in muscle sarcomeres (Nishikawa, 2020). Titin is the largest known protein, extending from the Z-line to the M-band (Bang et al., 2001; Linke, 2018). In the Z-line and A-band, titin is bound to the thin and thick filaments, respectively. In contrast, I-band titin is a freely extensible, viscoelastic spring, that is predominantly comprised of a relatively compliant proximal Ig domain region, a stiff PEVK region, (named for its predominant residues), and an N2A region that spans between the two springs (Linke et al., 1998a,b). Because of its location within the sarcomere, titin is responsible for nearly all longitudinal force in relaxed myofibrils (Granzier and Irving, 1995) and was suggested to function as a Ca^2+^-dependent spring in active muscle (Leonard and Herzog, 2010; Nishikawa et al., 2012; Linke, 2018). Recent work has demonstrated that the N2A region of titin binds to actin in a Ca^2+^-dependent manner (Dutta et al., 2018; Nishikawa et al., 2019), which would shorten titin’s free length and allow for stretch of only the stiffer PEVK region in active muscle. The *mdm* mutation in mice produces a small deletion in N2A titin (Garvey et al., 2002), which profoundly reduces active muscle stiffness (Powers et al., 2016; Hessel and Nishikawa, 2017; Monroy et al., 2017), leading to a reduction in both force enhancement with stretch and force depression with shortening (Tahir et al., 2020), apparently by preventing N2A titin–actin binding (Dutta et al., 2018; Nishikawa et al., 2019).

Many previous experiments have evaluated force during isovelocity stretch-hold (i.e., force enhancement) or shorten-hold (i.e., force depression) contractions (recently reviewed by Herzog, 2018; Chen et al., 2019). While these experiments provide important details about the history-dependence of force during and after isovelocity stretch and shortening, they fail to capture the history-dependent properties of muscles during SSCs (Seiberl et al., 2015b; Hahn and Riedel, 2018; Fukutani and Herzog, 2020b), in which energy stored during stretch can be recovered during shortening to increase net work per cycle (Hahn and Riedel, 2018; Fukutani and Herzog, 2020b). Most previous *ex vivo* studies using SSCs typically varied the velocity or amplitude of length changes (Askew and Marsh, 1997, 1998), but only rarely have such studies examined the effects of cross-bridge inhibition on muscle force and work (Fukutani and Herzog, 2020b). While it is now widely believed that cross bridges contribute relatively little to energy storage during active stretch (Linari et al., 2003; Pinniger et al., 2006), the mechanisms and extent of contributions from non-cross bridge elements such as titin to energy storage and recovery in SSCs remains to be elucidated. Based on the above arguments, it seems reasonable that a Ca^2+^-activated, non-cross bridge, viscoelastic element, specifically titin, contributes to muscle force and work during SSCs.

In this study, we used a multi-level, *ex vivo* approach to test broadly for contributions of Ca^2+^-activated, non-cross bridge, viscoelastic elements to SSCs in two different experimental preparations and muscles: whole soleus muscles and permeabilized psoas fibers. In whole muscle experiments, we used doublet stimuli at different phases of SSCs in wild type and *mdm* muscles to test for a role of titin in activation-and phase-dependence of peak force and work. In permeabilized psoas fibers, we used the myosin inhibitor, BDM to investigate contributions of Ca^2+^-sensitive non-cross bridge elements at different activation levels. By taking similar approaches that vary activation in the two preparations, we aim to further our understanding of the role of titin in regulating muscle force and work during SSCs.

For whole muscles, our strategy was to use doublet stimuli at varying phases of SSCs in wild type and *mdm* soleus muscles to test whether titin contributes not only to muscle force and work, but also to doublet potentiation as well as to the phase-dependence of activation, which feature prominently in the biomechanics of *in vivo* movements (Dickinson et al., 2000; Seiberl et al., 2015a; Paternoster et al., 2016). Doublet stimuli, when added to a train of low-frequency stimuli, potentiate muscle force (Burke et al., 1970, 1976) for up to hundreds of milliseconds after the single extra stimulus (Sandercock and Heckman, 1997). Although few previous studies have investigated doublet potentiation in SSCs, a single stimulus has been observed to increase work per cycle by up to 50% (Stevens, 1996). While this property of muscle has been known for more than 50 years, the underlying mechanisms are not well explained by the sliding filament-swinging cross bridge theory of muscle contraction (Burke et al., 1970; Sandercock and Heckman, 1997; Binder-Macleod and Kesar, 2005; Nishikawa et al., 2018). Several studies have shown that the Ca^2+^ transient associated with the doublet stimulus returns to control levels within ~25 ms (Abbate et al., 2002; Cheng et al., 2013; Bakker et al., 2017), but the increase in muscle force that persists for hundreds of milliseconds cannot be explained by current theories (Burke et al., 1970; Binder-Macleod and Kesar, 2005; Nishikawa, 2018). Previous studies have suggested that, similar to invertebrate “catch” (Butler and Siegman, 2010), engagement of a Ca^2+^-activated elastic element could potentially explain the sustained force at low [Ca^2+^] following doublet stimulation (Parmiggiani and Stein, 1981; Sandercock and Heckman, 1997; Binder-Macleod and Kesar, 2005; Nishikawa, 2018). By varying the phase of doublet stimulation in SSCs, we also investigated a potential role for titin in the phase-dependence of activation (Dickinson et al., 2000; Ahn, 2012).

For permeabilized muscle fibers, we developed a different strategy to investigate the relative contributions of Ca^2+^-sensitive cross bridges and non-cross bridge elements to muscle force and work in SSCs. Many previous studies have reported that, as activation increases in SSCs, peak force during lengthening also increases, which correlates with increased positive work during the subsequent shortening phase (Seiberl et al., 2015b; Fortuna et al., 2017; Fukutani and Herzog, 2020a). Of note, treatment of single muscle fibers with 10 mM BDM (~50% maximum activation level) enhanced shortening work during SSCs relative to a shortening-only contraction (Fukutani and Herzog, 2020b), suggesting that a Ca^2+^-sensitive, non-cross bridge viscoelastic element remains engaged during myosin inhibition. Here, we extend these observations to a wide range of concentration of calcium ions (pCa)- and BDM-regulated activation levels (15–100%). If a Ca^2+^-sensitive, non-cross bridge viscoelastic element remains engaged during myosin inhibition, then we expect that peak force and work during SSCs will be greater in BDM-treated than in Ca^2+^-activated fiber bundles matched to the same level of activation. We measured force and work of permeabilized fiber bundles during SSCs across differing levels of activation, achieved by varying the pCa and the myosin inhibitor, 2,3-butanedione monoxime (BDM) in a series of activating solutions. BDM reduces the proportion of actin-bound myosin heads (Griffiths et al., 2006), and therefore the isometric force of Ca^2+^-activated fibers. We matched activation levels of mouse psoas fiber bundles activated using solutions with different pCa to similar bundles activated using varying doses of BDM at pCa = 4.2. By comparing BDM-controlled and pCa-controlled fibers at a given activation level, we sought to determine whether force and work during SSCs were produced by cross bridges alone, or whether there was evidence for a contribution of additional Ca^2+^-dependent, viscoelastic elements.

In summary, our multi-level approach focuses on varying activation levels in SSCs: using pCa and BDM in psoas fiber bundles, and changing stimulation patterns (i.e., doublets and stimulation phase) in whole soleus muscles. At the whole muscle level, we hypothesize that titin contributes to doublet potentiation of muscle force and work during SSCs, and that the *mdm* mutation reduces not only the doublet effect but also the phase-dependence of activation on muscle force and work. At the level of permeabilized fibers, we predicted that, if only cross bridges contribute to force and work, and then there should be no difference in SSCs between BDM-controlled vs. pCa-controlled fibers across activation levels. However, if Ca^2+^-dependent viscoelastic elements also contribute, then BDM-controlled fibers should show greater force and work than pCa-controlled fibers.

## Materials and Methods

Animal use was approved by the Institutional Animal Care and Use Committees of the University Clinic Muenster (LANUV NRW, 81-02.04.2019.A472), Northern Arizona University (NAU IACUC Protocol #18-002), and the Claremont Colleges (CC IACUC Protocol #017-003). Adult mice were euthanized by an isoflurane gas overdose, cervical dislocation, and cardiac puncture. For whole muscle studies, wild type and homozygous recessive (*mdm*) B6C3Fe a/a-Ttn *^mdm^*/J mice from a C57BL/6 J background were obtained from a breeding colony at Northern Arizona University. Homozygous *mdm* mice can be identified by their small size, stiff gait, and hunchback posture at 10–12 days of age (Garvey et al., 2002; Lopez et al., 2008). Fiber studies were conducted on wild type mice with the same genetic background (C57BL/6 J) from a colony at University of Muenster.

### Whole Muscle Experiments

Whole muscle experiments were conducted on 11 wild type and 18 *mdm* soleus muscles from age-matched mice of both sexes (average wild type age = 44.4 ± 2.0 days; average *mdm* age = 49.6 ± 2.9 days, *p* = 0.12). Because wild type mice were larger in body size than *mdm* mice, muscle mass, optimal length (L_0_), and maximum isometric force were significantly greater in wild type compared to *mdm* soleus (Table 1), as reported previously (Taylor-Burt et al., 2015; Hessel and Nishikawa, 2017; Monroy et al., 2017; Hessel et al., 2019; Tahir et al., 2020). Maximum isometric stress was also lower in *mdm* muscles, likely because titin transmits cross bridges forces from the A-band to the Z-disk (Horowits et al., 1986; Nishikawa, 2020). Neither Ca^2+^ sensitivity nor the force-velocity relationship differs between genotypes (Hessel and Nishikawa, 2017; Tahir et al., 2020). To account for differences in length and stress, muscle length was normalized to optimal length (L_0_) and relative differences in stress were compared between genotypes.

**Table 1:** Physiological characteristics of wild type and *mdm* soleus muscles.

	*mdm*	Wild type
Body mass (g)	7.5 ± 0.30^*^	23.4 ± 0.49
Muscle mass (mg)	1.96 ± 0.53^*^	9.3 ± 0.67
L_0_ (mm)	7.13 ± 0.26^*^	11.27 ± 0.33
Max isometric force (N) ForForcewFocforce (N)	0.008 ± 0.006^*^	0.14 ± 0.01
P_0_ (Ncm^−2^)	4.1 ± 1.2^*^	15.6 ± 1.53

Data expressed as mean ± SEM, *mdm*: *n* = 18, wild type: *n* = 11.

* indicates *p* < 0.05.

To obtain force and length measurements during SSCs, the distal end of each muscle was attached to an inflexible hook, and the proximal end was attached to a dual servomotor muscle lever (Aurora Scientific, Inc., Series 300B, Aurora, ON, Canada). A custom LabVIEW (National Instruments Corp., Austin, TX, United States) program was used to control the muscle lever and record force, length and time at a sampling rate of 4 kHz. Muscles were kept in a Krebs–Henseleit bath (in mM: 137 NaCl, 5 KCl, 1 NaH_2_PO_4_, 24 NaHCO_3_, 2 CaCl_2_, 1 MgSO_4_, and 11 dextrose, pH 7.4), buffered with 95% O_2_ and 5% CO_2_. Because SSC experiments can fatigue muscles quickly, whole muscles were maintained at a constant temperature (21–23°C), at which the maximum isometric tetanic force remains stable for several hours and within 10% of the maximum isometric stress at normal body temperature of 37°C (James et al., 2015).

Maximum isometric tetanic force was measured from soleus muscles stimulated at 70–80 Hz for 800–1,500 ms at supramaximal voltage (Monroy et al., 2017). Periodically, the maximum isometric tetanic force was measured, and a muscle was removed from the analysis if force dropped by more than 10% from the initial value. At the start of each SSC experiment, muscles were set to optimal length (L_0_), defined as the length at which maximum isometric twitch force was produced. The maximum isometric stress (P_0_, Ncm^−2^) was determined by dividing maximum isometric force by the physiological cross-sectional area, calculated using standard methods (Hakim et al., 2013; Monroy et al., 2017).

**Figure 1:**
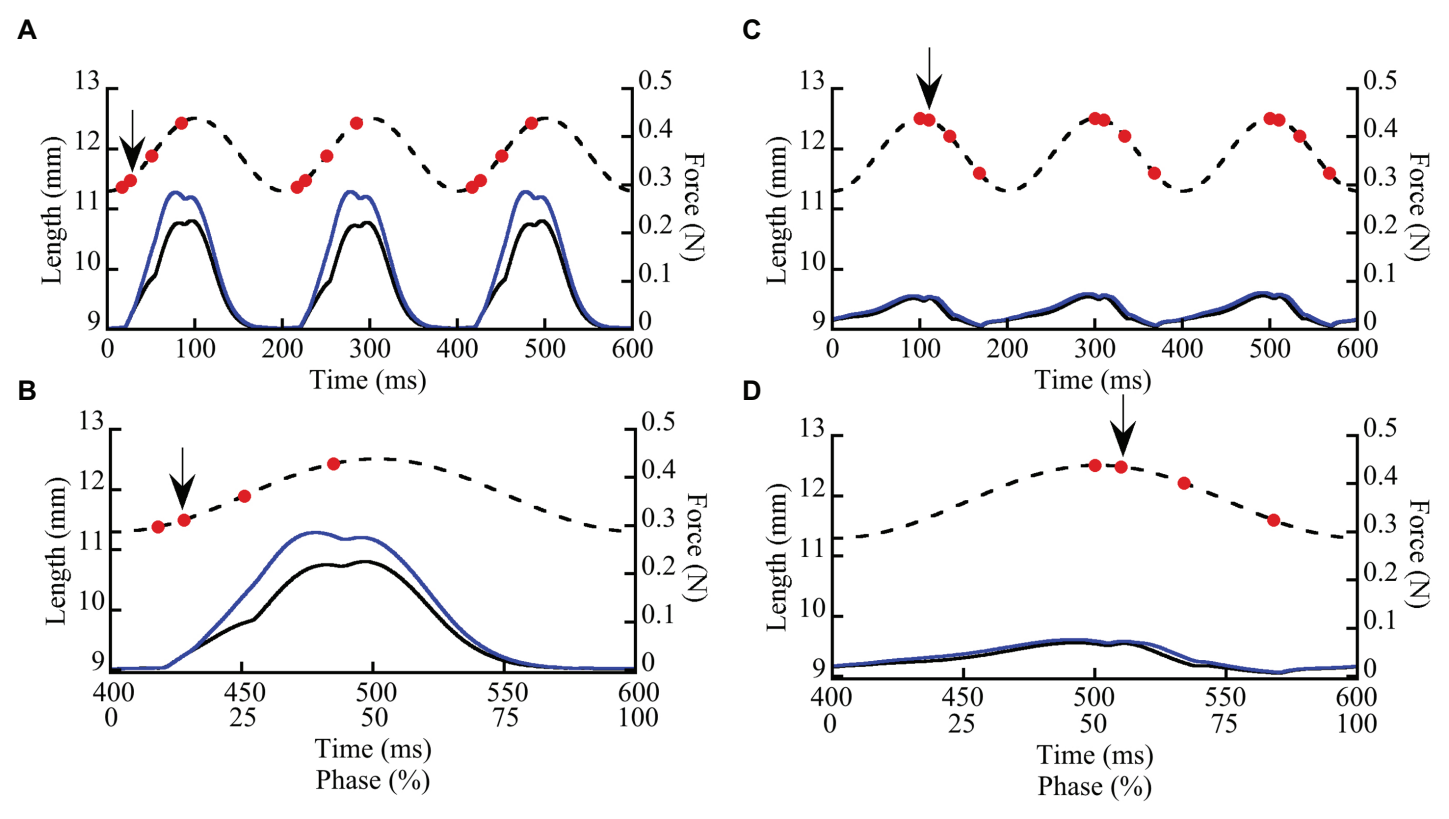
Representative stretch-shortening cycles (SSCs) from whole wild type soleus muscles stimulated at two phases of activation (8.3 and 50%). Muscles were subjected to 3–4 SSCs at strain amplitude of ±5% L_0_ and a frequency of 5 Hz (200 ms/cycle). In this example, a muscle was stimulated at a phase of 8.3% **(A,B)** when the muscle was lengthening and at a phase of 50% **(C,D)** at the onset of muscle shortening. **(A,C)** illustrate three successive cycles. **(B,D)** show the third cycle from each trial, as well as the relationship between time (ms) and phase (0–100%). Dashed lines show length vs. time and solid lines show force vs. time. Muscle force (solid lines) is shown for SSCs with (blue lines) and without (black lines) a doublet stimulus added 10 ms after the first stimulus in each cycle. Red dots indicate individual stimuli, the arrow indicates the doublet.

We measured the effects of doublet potentiation at different phases during SSCs in wild type and *mdm* soleus muscles to determine not only whether titin contributes to muscle force and work, but also to the activation- and phase-dependence of these quantities. Small amounts of doublet potentiation in fast-twitch muscles stimulated at nearly the maximum frequency can be explained by an increased number of cross bridges (Bakker et al., 2017), but not the larger and longer lasting effects that are observed at lower stimulation frequencies (see e.g., Abbate et al., 2002). Long-lasting doublet potentiation in soleus muscles that persists for >200 ms also is not readily explained by cross-bridge models (Sandercock and Heckman, 1997). By measuring muscle work during SSCs in wild type and *mdm* soleus muscles with and without doublet stimulation, we can determine whether titin likely contributes to doublet potentiation of muscle force and work. Muscles were subjected to ±5% L_0_ SSCs at a frequency of 5 Hz for a total of four cycles with and without doublet stimulation (Figures 1A,C). The cycle phase is defined here as the percent of an individual stretch-shortening cycle (SSC), where a phase of 0% denotes a muscle at its shortest length (−5% L_0_) at the onset of lengthening, a phase of 50% denotes a muscle at its longest length (+5% L_0_) at the onset of shortening, and the cycle ends at a phase of 100% (Figures 1B,D). Muscles were stimulated submaximally at 30 Hz for 100 ms.

To investigate the phase dependence of doublet stimulation, activation was initiated at six different phases within the SSC (8.3, 25, 33.3, 50, and 83.3%), which determined muscle length at the onset of stimulation as well as the direction of the work loop (clockwise for positive work and counter-clockwise for negative work). Each activation onset was repeated with and without a single doublet stimulus. Doublet stimuli were added 10 ms following the initial stimulus, for a total of three or four stimuli per cycle (see Figure 1; doublet stimulus indicated by black arrow). Passive and maximally stimulated (i.e., 75–80 Hz stimulus frequency) work loops were also obtained at each phase of stimulation. Muscles were rested 5–7 min between trials to minimize fatigue. For each trial, peak stress (Ncm^−2^) and mass-specific net, negative, and positive work were measured from the third cycle of each trial (James et al., 1995) using MATLAB (2019) [version 9.7.0. (R2019b) Natick, Massachusetts: The MathWorks Inc.].

### Permeabilized Fiber Bundle Experiments

Permeabilized (“skinned”) fibers were prepared from wild type mouse psoas muscles (*n* = 8 mice, five male/three female, age range 3–6 months) using standard glycerol techniques (Hessel et al., 2019). Extracted muscles were permeabilized and stored in a relaxing solution [in mmol l^−1^: potassium propionate (170), magnesium acetate (2.5), MOPS (20), K_2_EGTA (5), and ATP (2.5), pH 7.0] for 12 h at 4°C, then moved to a relaxing: glycerol (50:50) or rigor: glycerol solution [KCl (100), MgCl_2_ (2), EGTA (5), Tris (10), DTT (1), 50% glycerol, and pH 7.0] at −20°C for a minimum of 3 weeks. To limit protein degradation, all solutions contained one tablet of protease inhibitor (Complete, Roche Diagnostics, Mannheim, Germany) per 100 ml of solution. On the day of experiments, permeabilized muscles were removed from the storage solution and vigorously washed in relaxing solution on ice. Small bundles of 3–10 fibers were separated from the muscle. The ends of the fibers were wrapped with fine sutures and placed in custom-made clips to secure the fibers in place. Fibers bundles were attached lengthwise to a piezomotor on one end and a force transducer on the other end *via* aluminum clamps (Scientific Instruments, Heidelberg, Germany). All mechanical experiments were performed at 21°C. Force data were recorded at 1,000 Hz. Each fiber bundle was suspended in a bath of relaxing solution and then transferred to other baths as needed. Sarcomere length (SL) was measured by laser diffraction and initially set to an average length of 2.6 μm SL. Length changes were accomplished by computer-control of the length motor. Force was set to slack length = 0 mN for each sample. Total force is a combination of passive and active force components.

To separate the effects of [Ca^2+^] on cross bridge and non-cross bridge elements, we conducted mechanical experiments on fiber bundles activated to different levels by either: (1) changing the level of [Ca^2+^] (expressed as pCa = −log[Ca^2+^]); or (2) reducing cross bridge force production using the myosin inhibitor BDM at supramaximal pCa = 4.2. Different activating solutions were prepared from the same stock activating solution [in mmol l^−1^: potassium propionate (170), magnesium acetate (2.5), MOPS (10), ATP (2.5), and pH 7.0] by adding CaEGTA and K_2_EGTA in different proportions to obtain different pCa levels. For pCa-controlled experiments, we prepared activating solutions with a final pCa range between 4.2 and 6.2 to achieve a range of isometric forces (confirmed during pilot tests). For the BDM-controlled solutions, we added BDM to supramaximal activation solutions (pCa 4.2) to final concentrations between 0 and 30 mM BDM, to achieve a range of isometric forces from 100 to ~15% of maximum isometric force. The goal was to perform SSCs on individual fiber bundles over as a wide range of activation levels as possible to reduce variability associated with comparing data across different samples. Fibers were subjected to either the BDM- or the pCa- controlled protocols, but not both; we found that some level of force-reduction remained after BDM treatment, even with thorough washing.

**Figure 2:**
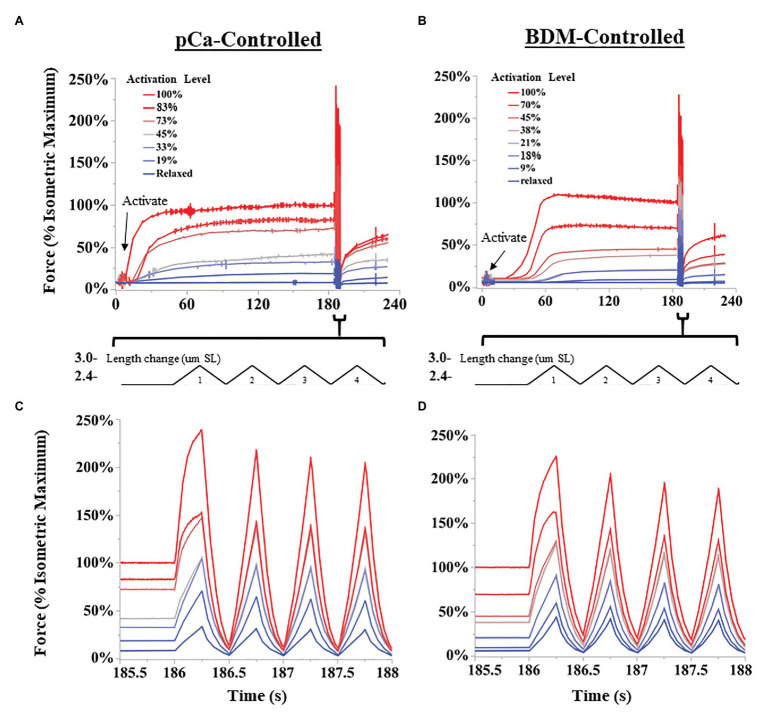
Representative SSC experiments from permeabilized fiber bundles. Activation levels were controlled either by decreasing the concentration of calcium ions (pCa; **A,C**), or by 2,3-butanedione monoxime (BDM)-induced myosin inhibition **(B,D)**. Length changes were performed using a “triangular” length trajectory between 2.4 and 3.0 μm SL at 2 Hz. **(A,B)** show all force trials collected for a representative fiber bundle for pCa-controlled and BDN-controlled fibers, respectively. Bundles were activated until force leveled off, and then subjected to multiple SSCs. **(C,D)** show the first four cycles at a higher time resolution for pCa and BDM trials, respectively.

The SSC protocol was as follows (Figure 2): muscle fibers were either kept relaxed or isometrically activated at 2.6 μm SL until isometric force was reached (1.5–4 min), and then cycled through eight successive triangular SSCs, from 2.6 to 3.0 μm SL at 2 Hz (1.6 μm SL s^−1^). After completion of SSCs, fibers were held at constant length (2.6 μm SL) for 30 s, and finally deactivated in relaxing solution. All fibers were tested in the following order: (1) Passive fibers were moved through the SSC protocol in a relaxed state 3–5 times to pre-stress the sample, which makes the force data more consistent (Li et al., 2020; Rivas-Pardo et al., 2020). The final passive SSC protocol was saved and used as the relaxed state. (2) Fibers were maximally activated (pCa 4.2) and moved through the SSC protocol. (3) The SSC protocol was repeated from lowest to highest activating solutions (increasing activation level for both BDM and pCa trials), up to seven different activating solutions per fiber, with at least 3 min rest in fresh relaxing solution between trials. This order was followed to maximize the number of tests that could be performed on a single fiber bundle before the force it produced dropped below 85% of its original maximum force, after which the fiber bundle was no longer used in experiments. We chose to use seven trials per sample after an extensive pilot study in which we subjected fiber bundles to various levels of pCa and measured changes in force over time. At certain submaximal activation levels, we found that fiber bundles could be activated up to 20 times before forces dropped below 85% of starting values. The conservative seven-trial cutoff was chosen to ensure that most fibers would be able to complete the experiment with minimal decrease in force. For each fiber bundle, the activation level (%) was calculated as the isometric force prior to the SSC divided by the maximum isometric force of the same fiber bundle at pCa = 4.2.

Generally, activation led to a typical leveling-off of isometric force over time, but the time to reach maximum force increased as activation level decreased (see Figures 2A,B). Some experiments at the lowest activation levels (>25 mM BDM; > pCa 6.1) required up to 4 min to develop maximum isometric force. Therefore, we used relatively long isometric activations before SSCs in all trials. After each trial, the fibers were visually assessed for damage using a 10x dissection microscope. We excluded the data and terminated the experiment if fibers tore, or if the passive force of relaxed fibers changed after the SSC protocol by >5%. In the present study, 12/43 fiber bundles were discarded. To compare across different fiber bundles, force data (mN total force) were normalized to stress (mN*mm^2^) using standard methods (Hessel et al., 2019). Length data were normalized to strain using the initial length of 2.6 μm SL. Peak force and specific work were normalized to the fiber’s isometric force at pCa 4.2 (reference trial). In total, we collected contraction trials from 16 and 15 fiber bundles in BDM and pCa-controlled protocols, respectively.

### Statistical Analysis

Statistical analyses were conducted using the lme4, lmtest, tidyverse, car (Fox and Weisberg, 2010), and MASS packages in R Studio statistical software (R Studio Team, 2020), Microsoft Excel (v11, Microsoft Inc., Seattle, WA, United States), and JMP (JMP Pro 14, SAS Institute). Alpha values were set at 0.05. Data were best Box-Cox transformed to meet assumptions of normality and homoscedasticity when necessary. Data are presented as mean ± SE (sem).

#### Whole Muscle Experiments

To assess effects of genotype and phase of stimulation on SSC variables, we used a linear mixed model with genotype and phase as main effects, the genotype × phase interaction, and individual nested within genotype as a random effect. The dependent variables were the differences between the control and doublet conditions for peak stress, net work, positive work, and negative work per cycle. Because peak stress, net, and negative work changed curvilinearly with phase of stimulation, a quadratic term for phase and the interaction between the quadratic term and genotype was added to the model. A log likelihood ratio test was used to determine the significance of each independent variable. The quadratic term was not significant and therefore not included in the model describing the difference in positive work across genotypes and phase of activation. Because maximum isometric stress was significantly smaller in *mdm* muscles, the dependent variables were recalculated as the % difference relative to the control and the analysis was repeated. All variables changed curvilinearly with phase of stimulation, therefore a quadratic term for phase and the interaction between the quadratic term and genotype was also included in the model.

#### Fiber Bundle Experiments

We compared four SSC variables across activation levels and between pCa-controlled and BDM-controlled conditions. From the stress-time traces, we calculated the forces at the longest length (peak force) during each SSC. Negative (lengthening phase), positive (shortening phase), and net (total) specific work per cycle were also calculated for each SSC. For clarity, and because trends are similar between SSCs, we only present analysis of SSC 1 in the main text, while data for all SSCs are available in supplemental information (see Supplementary Figure S1). We plotted relative values of force and specific work against activation level.

To assess the effects of condition and force on all four variables, we ran an analysis of covariance (ANCOVA) in JMP. The model included condition (pCa vs. BDM) as the main effect, activation level as the covariate, the condition by activation level interaction, and individual nested within condition as a random effect. To compare fibers activated with Ca^2+^ or Ca^2+^ plus BDM, the ANCOVA analyses were conducted for peak force and work in SSCs from 52 pCa and 76 BDM-controlled fibers across activation levels (% isometric force before the SSCs).

## Results

### Whole Muscle Experiments

**Figure 3:**
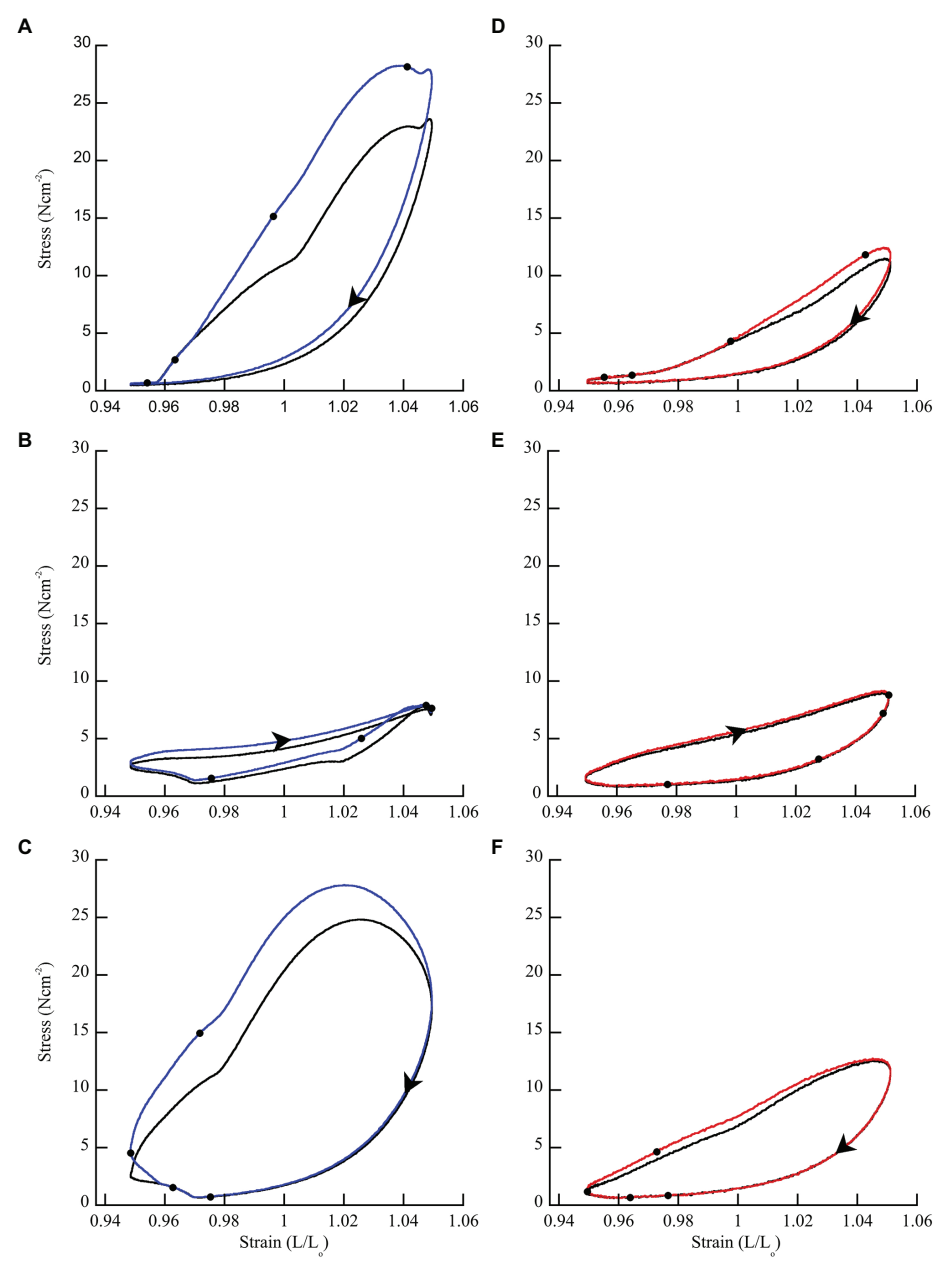
Representative work loops from wild type and *mdm* soleus muscles stimulated with (blue = wild type; red = *mdm*) and without (black) a doublet. **(A,D)** Phase = 8.3% when the muscle is lengthening. **(B,E)** Phase = 50% when the muscle is shortening. **(C,F)** Phase = 83.3% when the muscle is shortening followed by lengthening. The effect of doublet stimulation on work per cycle (area within the work loop) changes with the phase of activation and is greatest when stimulation occurs during lengthening (phase < 50%). Black dots indicate individual stimuli. Arrows indicate the direction of a work loop.

In general, when wild type muscles were activated with doublet stimulation during lengthening (phase < 50%) force and work increased, and the increase persisted throughout shortening for 100 ms after stimulation ceased (Figure 3A). When activated during shortening (phase = 50%), doublet stimulation resulted in smaller changes in positive and negative work (Figure 3B). Activation at the end of shortening and through the beginning of lengthening (phase > 70%) caused a large increase in negative work but small change in positive work (Figure 3C). Across all phases of activation, *mdm* muscles exhibited little doublet potentiation of force or work (Figures 3D–F).

**Figure 4:**
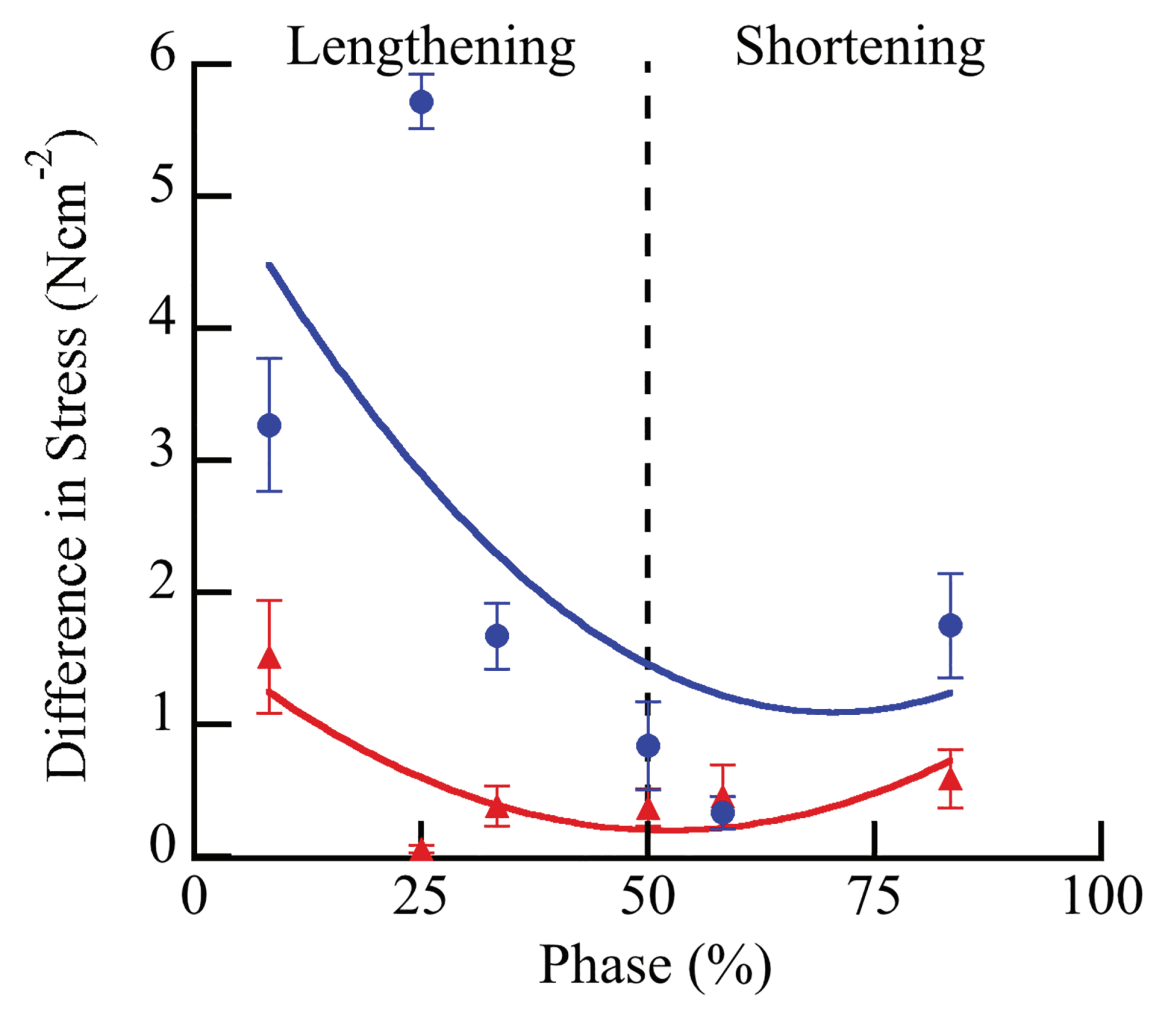
Differences in peak stress with vs. without doublet stimulation across phase of stimulation in wild type (blue) and *mdm* (red) soleus. Doublet potentiation of peak stress is reduced in *mdm* muscles compared to wild type at all phases of activation (*F* = 52.3, *p* < 0.0001). Phase = 0% indicates a muscle at its shortest length at the onset of lengthening and phase = 50% indicates a muscle at its longest length just prior to shortening. Data represent means ± SE, *N* = 28 individuals, 112 trials.

There was a significantly greater increase in peak stress with doublet stimulation in wild type than in *mdm* muscles (Figure 4; *F* = 52.3, *p* < 0.0001). There was also a significant interaction between the quadratic term and genotype, indicating a difference in curvature of the stress-phase relationship between genotypes (Figure 4; *F* = 13.3, *p* = 0.0004). In wild type soleus, the greatest increase in peak stress occurred at phase = 25%. However, only two muscles were stimulated at this phase, so the mean value may be an over-estimate. For all other phases, at least seven muscles were included in the analysis. In general, the greatest increase in peak stress with doublet stimulation occurred when muscles were activated during lengthening and the smallest increase occurred during shortening. A separate analysis of only *mdm* data showed no effect of phase (*F* = 0.2, *p* = 0.65) or the quadratic term (*F* = 0.04, *p* = 0.85) on peak stress in *mdm* muscles (Figure 4). Similarly, the relative increase in peak stress with doublet stimulation was greater in wildtype compared to *mdm* muscles (Supplementary Figure S1; Supplementary Table S1; *F* = 23.7, *p* < 0.0001). However, there was no significant interaction between genotype and the quadratic term, indicating that the relationship between the relative increase in peak stress and phase did not differ between genotypes (*F* = 0.49, *p* = 0.48).

**Figure 5:**
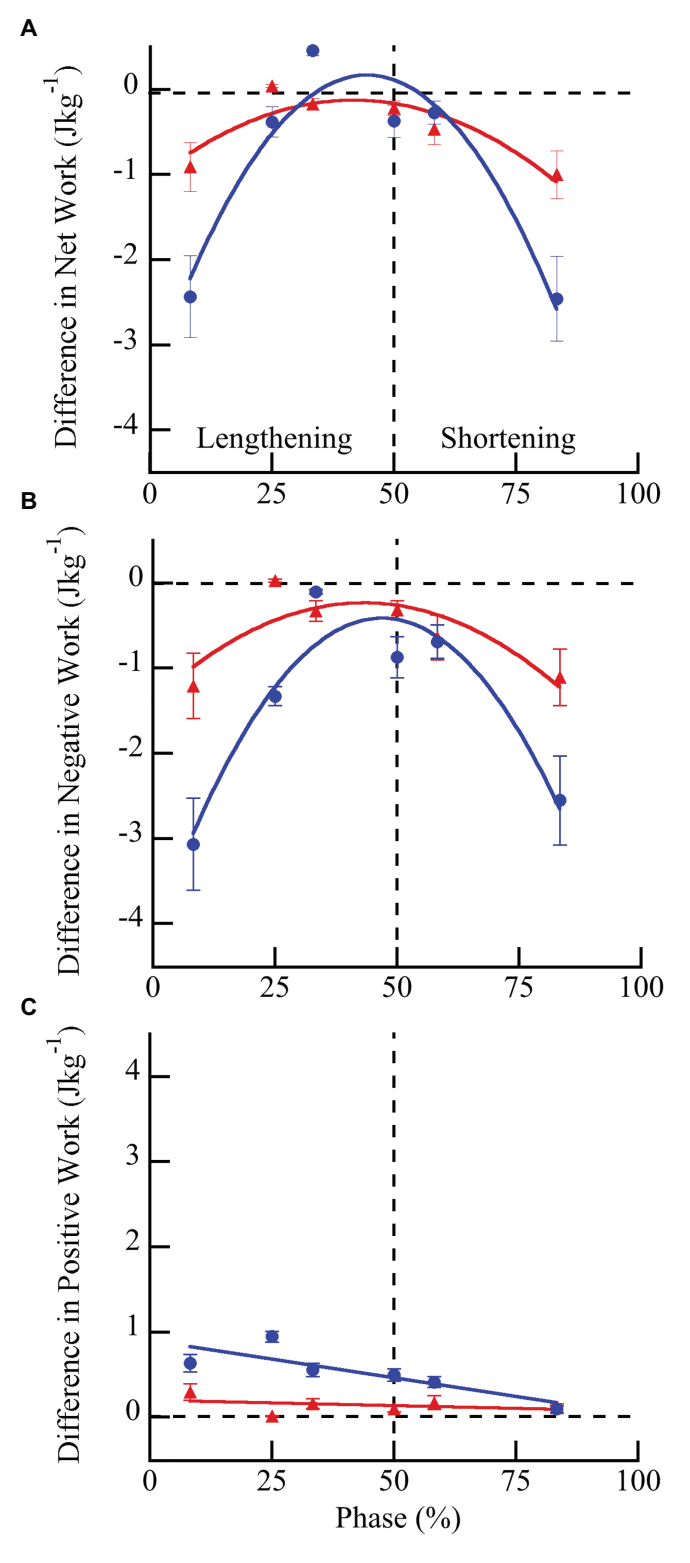
Differences in net **(A)**, negative **(B)**, and positive **(C)** work per cycle with vs. without doublet stimulation across phase of activation in wild type (blue) and *mdm* (red) muscles. The greatest increase in work with doublet stimulation occurred when muscles were activated during lengthening and the smallest increase occurred when muscles were activated during shortening. Wild type muscles exhibited a significantly greater increase in net (*F* = 43.84, *p* < 0.0001), negative (*F* = 14.51, *p* = 0.007), and positive (*F* = 50.43, *p* < 0.0001) work than *mdm* muscles across all phases of activation. Phase = 0% indicates a muscle at its shortest length at the onset of lengthening and phase = 50% indicates a muscle at its longest length just prior to shortening. Data represent means ± SE, *N* = 28 individuals, 112 trials.

Wild type muscles exhibited a significantly greater increase in net (*F* = 43.84, *p* < 0.0001), negative (*F* = 14.51, *p* = 0.0007), and positive work (*F* = 50.43, *p* < 0.0001) with doublet stimulation compared to *mdm* muscles (Figure 5). Similar to peak stress, the greatest increase in work occurred when muscles were activated during lengthening and the smallest increase occurred when muscles were activated during shortening. Wild type muscles exhibited a 3-fold greater increase in negative work with doublet stimulation than *mdm* muscles, and there was a significant interaction between the quadratic term and genotype on both net (*F* = 49.81, *p* < 0.0001) and negative work (*F* = 40.87, *p* < 0.0001), indicating that the curvature of the relationship between these variables and phase differed between genotypes (Figures 5A,B). Positive work decreased linearly with phase and the slope of this relationship was five times greater in wild type than *mdm* muscles (Figure 5C; *F* = 20.88, *p* < 0.0001). Relative changes in net, negative, and positive work were similar to absolute differences (Supplementary Figure S2; Supplementary Table S1). During lengthening, the relative increases in both negative and positive work were more than 20% greater in wild type compared to *mdm* muscles. Interestingly, at phase = 33%, when wild type muscles were stimulated at the end of lengthening and through the beginning of shortening, there was a more than 2-fold increase in positive work with doublet stimulation, causing net work to shift from negative to positive and to increase by more than 150% (Supplementary Figure S2).

### Permeabilized Fiber Bundle Experiments

Initial total isometric stress of fiber bundles in pCa 4.2 solution with no BDM was similar (ANOVA; *F* = 0.07; *p* = 0.79) between pCa-controlled (*N* = 15; force = 2.05 ± 0.24 mN; PCSA = 0.017 ± 0.008 mm^2^; stress = 116.60 ± 4.2 mN*mm^2^) and BDM-controlled fibers (*N* = 16; force = 2.24 ± 0.38; PCSA 0.019 ± 0.012 mm^2^; stress = 119.02 ± 7.9 mN*mm^2^), as expected. Furthermore, the initial isometric stress of relaxed fibers (passive, relaxing solution with no BDM at 2.6 μm SL) was also similar (ANOVA; *F* = 0.52; *p* = 0.48) between pCa-controlled (12.989 ± 9.4 mN*mm^2^) and BDM-controlled fibers (10.42 ± 5.9 mN*mm^2^).

**Figure 6:**
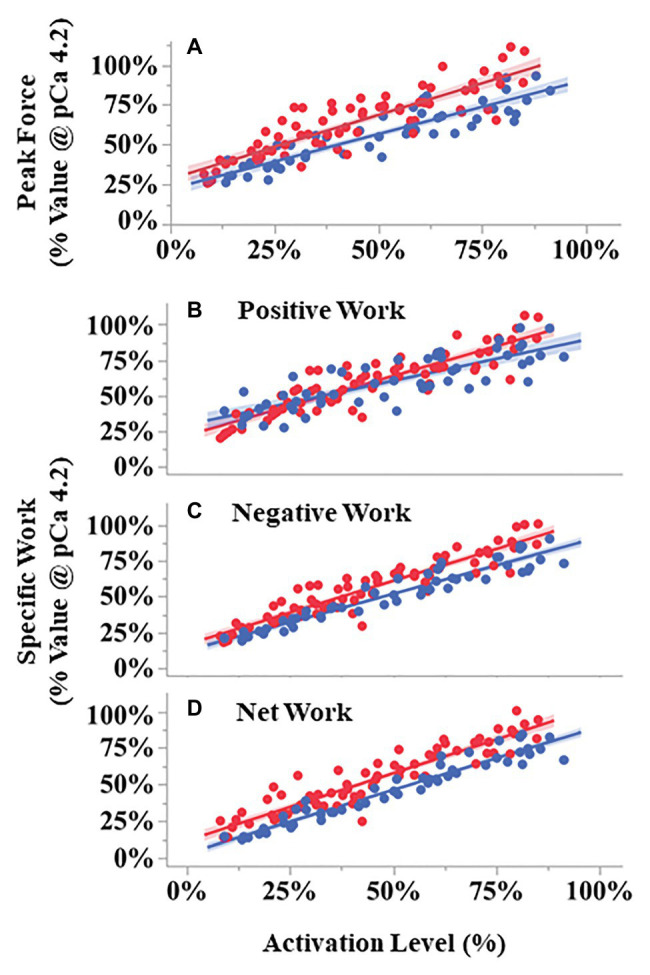
Peak force and work during SSCs vs. activation level for pCa- (blue) and BDM- (red) controlled fibers. All variables scaled linearly with activation level analysis of covariance (ANCOVA covariate effect < 0.001). Peak forces **(A)** were larger in BDM- vs. pCa- controlled trials across activation levels (*p* = 0.0014). Both negative (*p* < 0.001) and total work (*p* = 0.014) components were also larger in BDM vs. pCa controlled fibers. Compared to pCa-controlled trials, BDM-controlled trials had a modest but statistically significant steeper increase with activation level for positive work (**B**, ANCOVA interaction effect, *p* = 0.0072) and negative work (**C**, *p* = 0.031), but not for net work (**D**, *p* = 0.16). The 95% CIs are shown by the shaded regions around the lines. *N* = 15 pCa and 16 BDM experiments, *n* = 52 pCa and 76 BDM trials.

During the eight SSCs, the first SSC always produced the largest peak force (up to 250% maximal isometric force), with subsequent SSCs decreasing in force until SSC 5–8 when the force became approximately constant (Supplementary Figure S3). The relationship between peak force during the first SSC vs. activation level differed between pCa- and BDM-controlled fibers (Figure 6A). ANCOVA showed that peak force increased with increasing activation level for both conditions (activation level covariate *F* = 680.71, *p* < 0.001). Furthermore, compared to pCa-controlled fibers, the peak force of the BDM-controlled fibers was higher at a given activation level (condition *F* = 12.59, *p* = 0.0014) and trended toward a steeper slope than in pCa-controlled fibers (interaction *F* = 3.78, *p* = 0.055; Figure 6A).

The relationships between specific positive, negative, and net work per cycle vs. activation level also differed between pCa- and BDM-controlled fibers (Figures 6B–D). For all three work variables, there was a significant positive relationship between work and activation level (positive work *F* = 612.07, *p* < 0.001; negative work *F* = 1318.01, *p* < 0.001; net work *F* = 1399.58, *p* < 0.001). The relationship between positive work vs. activation level for BDM-controlled fibers produced a slightly steeper slope than for pCa-controlled fibers (interaction *F* = 7.53, *p* = 0.0072), and there was no significant difference between intercepts (condition *F* = 0.41, *p* = 0.53; Figure 6B). In contrast, the relationship between negative work vs. activation level produced steeper slopes (interaction *F* = 4.75, *p* = 0.031) and increased intercepts (condition *F* = 14.89, *p* < 0.001) in BDM-controlled fibers compared to pCa-controlled fibers (Figure 6C). Finally, the relationship between net work vs. activation level increased in BDM-controlled fibers compared to pCa-controlled fibers (condition *F* = 26.78, *p* < 0.001), but with similar slopes (interaction *F* = 1.90, *p* = 0.1709; Figure 6D). Taken together, our data indicate that specific negative and net work per cycle is greater in BDM- vs. pCa-controlled fibers.

## Discussion

The focus of our study was to conduct SSC experiments at two different levels of muscle organization to test the hypothesis that Ca^2+^-activated, non-cross bridge, viscoelastic elements contribute to muscle force and work during dynamic changes in length. We used whole muscle and single fiber preparations to ask whether the traditional sliding filament – swinging cross bridge theory alone can account for muscle force and work during SSCs. Together, both approaches provided support for the hypothesis that a Ca^2+^-dependent, non-cross bridge element, likely titin, contributes to force and work during SSCs.

### Whole Muscle Experiments

Three observations from whole muscle studies support the existence of non-cross bridge forces in SSCs. (1) In wild type muscles, potentiation of force and work persists for ~100 ms after doublet stimulation, long after ~25 ms [Ca^2+^] transients associated with a doublet stimulus have subsided (Bakker et al., 2017). (2) Adding a doublet stimulus to a submaximal pulse train during SSCs increased force and work in a phase-dependent manner, with stimulation during the eccentric phase producing the greatest increase. (3) The response to doublet stimulation was significantly reduced in *mdm* muscles with a small deletion in N2A titin (Garvey et al., 2002), showing almost no long-lasting effects throughout an SSC.

Results from the present study showed that doublet potentiation of force and work persisted throughout an SSC for 100 ms after stimulation ceased in wild type muscles. This observation corroborates previous findings which showed a rapid increase in force with doublet stimulation that can be sustained for long periods up to 250 ms under isometric conditions (Burke et al., 1970; Zajac and Young, 1980; Stein and Parmiggiani, 1981; Hennig and Lømo, 1987; Sandercock and Heckman, 1997; Abbate et al., 2002). Recent work by Bakker et al. (2017) showed that doublet stimulation increases intracellular [Ca^2+^] for the first 20–30 ms following stimulation. They suggested that the [Ca^2+^] transient might saturate the second Ca^2+^-binding site on TnC and lead to faster initiation of cross-bridge cycling, although this hypothesis remains to be tested experimentally. Nevertheless, the proposed increase in rate of cross-bridge cycling *via* saturation of TnC cannot account neither for increased force with doublet stimulation nor for persistence of the potentiated force for up to hundreds of milliseconds (Burke et al., 1976; Sandercock and Heckman, 1997) after the [Ca^2+^] level has returned to baseline levels (Abbate et al., 2002; Cheng et al., 2013; Bakker et al., 2017). Thus, while the rate of cross-bridge cycling may increase with a transient increase in [Ca^2+^] and therefore contribute to the rapid increase in force, neither the time constants of Ca^2+^ release and re-uptake nor the saturation of TnC are consistent with the sustained increase in force and work that occurs in response to doublet stimulation.

The increased effect of doublet stimulation during the eccentric phase of an SSC that we observed here matches previous work demonstrating that stretch of a muscle during doublet stimulation enhances force, whereas shortening abolishes the doublet effect (Sandercock and Heckman, 1997). Isometric studies have also shown that doublet potentiation is greatest at short muscle lengths on the ascending limb of the length-tension relationship and decreases as muscle length increases (Burke et al., 1976; Sandercock and Heckman, 1997). In addition, Stevens (1996) showed that adding a single doublet stimulus to an SSC can increase net work per cycle by more than 50%. The length-dependence of doublet potentiation is not easily explained by cross bridge models, but instead suggests the engagement of a Ca^2+^-dependent viscoelastic element (Binder-Macleod and Kesar, 2005) that stores elastic energy during stretch and releases energy during shortening, thereby increasing muscle force and work during eccentric SSCs (Nishikawa et al., 2018).

Interestingly, results from the present study also showed that doublet potentiation is markedly reduced in *mdm* muscles, which are characterized by a small deletion in the N2A region of titin (Garvey et al., 2002). The difference between wild type and *mdm* muscles was greatest when the muscles were activated during lengthening, where doublets increased negative, positive, and net work per cycle in wild type but not *mdm* soleus. These observations are consistent with other studies on *mdm* muscles, which demonstrated reduced force enhancement during active stretch and reduced force depression with active shortening (Tahir et al., 2020), as well as reduced net work during eccentric SSCs (Hessel and Nishikawa, 2017).

To our knowledge, this study is the first to test the hypothesis that titin contributes to doublet potentiation. Previous studies have suggested a role for elastic elements other than cross bridges (Stein and Parmiggiani, 1981; Sandercock and Heckman, 1997; Binder-Macleod and Kesar, 2005), but the mechanism for doublet potentiation remains unclear. Doublet potentiation is similar to “catch” in molluscan muscles, in that force is maintained for hours at low [Ca^2+^] (Butler and Siegman, 2010). The mechanism for molluscan catch has been shown to involve binding of twitchin, a giant sarcomeric protein related to titin, to thin filaments (Butler and Siegman, 2010), similar to N2A titin-actin binding which is thought to be responsible for titin activation (Dutta et al., 2018; Nishikawa, 2020). The addition of a doublet at the onset of a submaximal stimulus train causes a transient increase in [Ca^2+^], which likely increases in titin-actin binding in addition to cross bridge formation. However, once [Ca^2+^] returns to baseline levels between stimuli (~25 ms), cross bridges release but force remains elevated due to the much slower off rates (~200 ms) of titin-actin interactions (Dutta et al., 2018). At a cycle frequency of 5 Hz, titin would be expected to remain bound to actin for the entire duration of an SSC. How titin contributes to doublet potentiation of force during isometric contractions remains unclear because activation increases titin stiffness, thus strain is required to increase titin force. Hypotheses including titin winding on actin (Nishikawa et al., 2012) or a “sticky spring” (Rode et al., 2009) could potentially explain how titin contributes to force of isometrically contracting muscle but future work is needed.

This study is also the first to test the hypothesis that titin contributes to the phase-dependence of activation. When activated at its shortest length followed by stretch-shortening, a muscle behaves like a spring in which elastic energy stored during stretch enhances work during shortening. However, when activated at its longest length followed by shortening-stretch, a muscle behaves like a motor which produces positive work (Ahn, 2012). During *in vivo* movements, the phase-dependence of activation serves the important function of allowing a muscle to function in different ways for different tasks (Dickinson et al., 2000). The observation that the phase dependence of activation is reduced in skeletal muscles from *mdm* mice suggests a contribution for titin. We suggest that binding of N2A titin to actin at different muscle lengths depending on the phase of activation is a mechanism of phase-dependence. Titin-actin binding is thought to decrease titin free length and increase titin stiffness (Dutta et al., 2018; Nishikawa et al., 2019). Binding of titin to actin at differing distances from the Z-disk would have the effect of enhancing both the spring-like and motor-like properties of muscles depending on the phase of activation.

### Permeabilized Fiber Experiments

Using permeabilized fiber preparations and the myosin inhibitor BDM, we decoupled the relationships among [Ca^2+^], cross bridge formation, and force production by activating fibers at supramaximal [Ca^2+^] (pCa 4.2) but with reduced cross-bridge forces (Griffiths et al., 2006). We found that peak force, net work, and negative work (Figure 6) were greater in BDM compared to pCa-controlled fiber bundles at a given level of activation.

Early studies found that BDM decreased isometric force more than isometric stiffness. Using permeabilized rabbit psoas fibers, Seow et al. (1997) first reported that treatment with 3 mM BDM increased the stiffness to force ratio by 50%. The increased stiffness to force ratio was thought to be caused by an increase in the number of low-force (i.e., weakly bound) cross bridges that would contribute to stiffness but not active force (Bagni et al., 1992; Seow et al., 1997). However, subsequent analyses (Bagni et al., 1992, 2005; Griffiths et al., 2006) of the effects of BDM on thick and thin filament structure found that the fraction of attached cross bridges is similar between pCa- and BDM-controlled fibers at a given force. Therefore, the BDM-induced increase in the stiffness to force ratio has instead been attributed to the existence of Ca^2+^-sensitive, non-cross bridge viscoelastic elements (Griffiths et al., 2006; Colombini et al., 2016). BDM has also been used previously to investigate residual force enhancement after isovelocity stretch (Rassier and Herzog, 2005a,b). Using BDM to decrease active force, Rassier and Herzog (2005b) found that a 50% decrease in active force led to only a 15% drop in fiber stiffness and a non-statistically significant drop in the absolute residual force enhancement. These observations are also consistent with the existence of a Ca^2+^-sensitive, non-cross bridge, viscoelastic element that contributes to force enhancement after stretch.

One feature of SSCs is that the magnitude of peak force is positively related to force and work during the subsequent shortening phase (Seiberl et al., 2015b; Fortuna et al., 2017; Hahn and Riedel, 2018; Fukutani and Herzog, 2020a,b). A recent study (Fukutani and Herzog, 2020b) used permeabilized rabbit fibers to compare SSC mechanics between maximally activated fibers at pCa = 4.2 with or without 10 mM BDM, reducing the activation level by ~ 50%. The results showed that positive work produced during the shortening phase of SSCs was relatively larger in BDM-treated fibers, increasing from 169% of the isovelocity shortening work in 0 mM BDM to 205% in 10 mM BDM. In our study, we tested the effect of BDM on positive work during shortening across a range of activation levels from 15 to 100% maximum activation. We found that compared to pCa-controlled fibers, BDM treatment increased relative positive work only at high activation levels (>50% of the maximal isometric force). At low activation levels (<25% of maximal isometric force), positive work of BDM-treated fibers was slightly less than for pCa-controlled fibers (Figure 6B).

An often neglected feature of SSCs is the history-dependent property that peak force (Supplementary Figure S1) is greater during the first repetition in a series of multiple SSCs (see our Figure 2; Campbell and Moss, 2002). This property, called thixotropy (see review by Lakie and Campbell, 2019), is enhanced at lower activation levels and leads to changes in fiber stiffness over time during multiple SSCs. In a series of two consecutive SSCs in single fibers, Campbell and Moss (2002) found that fibers were more compliant during the second stretch phase than the first. However, if a delay was introduced between the two SSCs, then the fibers recovered a portion of their initial stiffness for the second SSC. For delays >1 s, fiber stiffness became identical between the two SSCs (Campbell and Moss, 2002). They also found that fiber stiffness during the shortening phase was less dependent on length history than during the lengthening phase. The time-dependent thixotropy of fibers during SSCs was not readily predicted by cross bridge models without the addition of a Ca^2+^-dependent parallel elastic element (Campbell and Moss, 2002).

While it is difficult to separate the contributions of cross bridge and non-cross bridge elements to force production during isometric contraction, Pinniger et al. (2006) conducted a clever set of experiments and used a cross bridge model to distinguish the relative contributions of cross bridge and non-cross bridge elastic elements to muscle force during stretch. They concluded that, after a small stretch of ~1.25–1.5% L_0_, the cross bridge contribution to force enhancement reaches a maximum value, as new detachment/attachment equilibrium is reached. During length changes >1.5% L_0_, force enhancement must be provided by non-cross bridge elements. Thus, the relative contribution of non-cross bridge elements increases with increasing stretch amplitude. During the SSC experiments reported here, we used strain amplitudes of 10% in whole muscles and 15% L_0_ in fiber preparations. By extrapolating from Pinniger et al. (2006), the force contribution of non-cross bridge elements is expected to be ~60 and ~70% of the total force rise for whole muscles and permeabilized fibers, respectively. However, these calculations are based on maximally activated fibers, where the number of cross bridges is maximized. The calculation therefore overestimates the contribution of cross bridges for the submaximal activation levels used in our SSC experiments. For example, at an activation level of 50%, the contribution of cross bridges to peak force would be reduced by half, suggesting that cross bridges might contribute as little as 15 or 20% to peak force in SSCs for permeabilized fibers or whole muscles, respectively.

### Titin as a Tunable Viscoelastic Element in Muscle Sarcomeres

Over the past 20 years, mounting evidence has suggested the existence of a Ca^2+^-sensitive, non-cross bridge, viscoelastic element in muscle sarcomeres. This element is engaged in the earliest stages of muscle activation at [Ca^2+^] levels below those required to activate thin filaments and permit cross bridge interactions (Bagni et al., 1994, 2002; Colombini et al., 2016). Single myofibril studies also demonstrate that the increase in stiffness with activation following active stretch persists for up to several minutes following deactivation (Joumaa et al., 2007, 2008). Recent estimates suggests that the Ca^2+^-sensitive non-cross bridge stiffness is 100 times greater than the passive stiffness in isometrically contracting single fibers (Powers et al., 2019). In their study, Powers et al. (2019) used high-frequency length oscillations (4 kHz) in isometrically contracting single muscle fibers to measure fiber stiffness, and found that the I-band stiffness increased by two orders of magnitude in isometrically contracting fibers compared to the passive stiffness of the same fibers at a sarcomere length of 2.7 μm SL.

Accumulating evidence further suggests that the giant sarcomeric titin protein is a Ca^2+^-sensitive, non-cross bridge, viscoelastic element, which functions as a tunable spring in active muscle (Leonard and Herzog, 2010; Nishikawa et al., 2012; Herzog, 2019; Nishikawa, 2020). Numerous recent studies support the hypothesis that N2A titin is a signaling hub and mechanical linker that binds to actin in the presence of Ca^2+^ (Nishikawa et al., 2020), thereby decreasing titin’s free length and increasing its stiffness (Dutta et al., 2018; Nishikawa et al., 2019, 2020; Nishikawa, 2020). Single molecule force spectroscopy experiments demonstrate that N2A-actin interactions are highly stable in the presence of Ca^2+^ (pCa < 5.0), and the off-rate is more than three times slower (4 v. 16 s^−1^) when Ca^2+^ is present (Dutta et al., 2018). Binding of N2A titin to actin brings the titin molecule closer to actin, which may increase the likelihood of hydrostatic interactions between PEVK titin and actin as well. PEVK interactions with actin resist stretch long after Ca^2+^ has decreased (Bianco et al., 2007).

The *mdm* mouse model is unique in exhibiting a small deletion in the N2A region of titin (Garvey et al., 2002). This region appears to be critical for mediating the Ca^2+^-dependent increase in titin stiffness that occurs upon muscle activation (Nishikawa, 2020; Nishikawa et al., 2020). Numerous studies demonstrate that active stiffness is reduced in muscles from *mdm* mice (Powers et al., 2016; Monroy et al., 2017). These muscles also exhibit reduced force enhancement following active stretch and reduced force depression following active shortening (Tahir et al., 2020), suggesting a role for titin in these history-dependent properties.

### Limitations

As is common practice, experiments were conducted at room temperature (25 and 21°C for whole muscle and fiber experiments, respectively) rather than body temperature (37°C) to reduce fatigue and fiber degradation (Hakim et al., 2013). Although muscle mechanics are altered by temperature (Stephenson and Williams, 1981, 1985; James et al., 2015; Ranatunga, 2018; Caremani et al., 2021), the effect is expected to be relatively small (~10%).

2,3-butanedione monoxime is only one way to inhibit cross bridge interactions, with other approaches including the use of different inhibitors such as N-benzyl-p-toluene sulphonamide (BTS; Pinniger et al., 2005), or chemical removal of troponin C (Joumaa et al., 2008). Because each method has its own limitations, it is important to repeat these fiber experiments with other cross-bridge inhibitors to compare with data from this study.

### Conclusion

The results from both whole muscle and permeabilized fibers in SSC experiments suggest the existence of a tunable viscoelastic element that enhances muscle force and stores strain energy during active stretch, and recovers stored energy during subsequent shortening to increase positive and net work per cycle. A tunable viscoelastic element such as titin can account for: (1) the persistence of force at low [Ca^2+^] with doublet potentiation; the phase- and length- and phase-dependence of doublet potentiation observed in whole wild type muscles and the absence of these effects in *mdm* muscles; and lastly, the increased peak force and work per cycle in both whole muscles and permeabilized fibers in SSC experiments. While it has been suggested that other non-cross bridge elements, such as nebulin (Kawai et al., 2018), microtubules (Kerr et al., 2015), and myosin binding protein C (Harris, 2021), may increase muscle stiffness during activation, no studies have demonstrated definitive mechanisms that could help to explain the increase in muscle force and work during dynamic length changes. So far, titin is the only known non-cross bridge viscoelastic element in muscle sarcomeres that has been shown to interact with Ca^2+^ and actin.

By combining SSC experiments in whole muscles and permeabilized fibers, we gain a better understanding of the Ca^2+^-dependent viscoelastic elements that contribute to the mechanical properties exhibited by active muscles during dynamic changes in length. Whole muscle studies have the advantage of simulating *in vivo* muscle activation and length changes, whereas in fiber preparations, we can quantify the relative contributions of cross bridge and non-cross bridge elements to dynamic force during SSCs. Both preparations showed an increase in peak force and work per cycle with increasing activation (adding a doublet in whole muscle experiments and decreasing pCa in permeabilized fibers), which was enhanced during lengthening contractions. It is important to point out that phase dependence could only be measured in whole muscle studies since muscles were activated with a duty cycle of 0.5, whereas fibers were activated with a duty cycle of 100% throughout the entire SSC protocol. Thus, work loops from fiber preparations more closely resembled eccentric work loops of whole muscles. Form these studies, it appears that perturbing activation in SSCs provides an experimental paradigm for quantifying the contributions of calcium-sensitive non-cross bridge viscoelastic elements to muscle force during dynamic length changes.

## Data Availability Statement

The raw data supporting the conclusions of this article will be made available by the authors, without undue reservation.

## Ethics Statement

The animal study was reviewed and approved by the Institutional Animal Care and Use Committees of Northern Arizona University (NAU IACUC Protocol #18-002), the Claremont Colleges (CC IACUC Protocol #017-003), and the University Clinic Muenster (LANUV NRW, 81-02.04.2019.A472).

## Author Contributions

JM and AH contributed equally to this work and were responsible for data collection and analysis. JM, AH, and KN all contributed to the conception and design of the experiments and interpretation of the data. All authors contributed to the article and approved the submitted version.

### Conflict of Interest

The authors declare that the research was conducted in the absence of any commercial or financial relationships that could be construed as a potential conflict of interest.
