# Supplementary material for: Non-cross Bridge Viscoelastic Elements Contribute to Muscle Force and Work During Stretch-Shortening Cycles: Evidence From Whole Muscles and Permeabilized Fibers
Source: Front Physiol. 2021 Mar 29;12:648019. doi: 10.3389/fphys.2021.648019 (PMC8039322; doi:10.3389/fphys.2021.648019)
Supplement: Supplementary file 1 [file Data_Sheet_1.pdf]

Supplementary Material

Supplementary Text

Because maximum isometric stress was significantly smaller in *mdm* muscles, the dependent variables (peak stress, net work, negative work, positive work) were recalculated as the % difference relative to the control trial without a doublet stimulus. We used a linear mixed effects model with genotype and phase as main effects, the genotype x phase interaction, and individual nested within genotype as a random effect to measure the effects of genotype and phase of stimulation on the dependent variables. All variables changed curvilinearly with phase of stimulation, therefore a quadratic term for phase ($phase^2$) and the interaction between the quadratic term and genotype (genotype x $phase^2$) were also included in the model. Results of this analysis are in Supplementary Table 1.

Because net work of wild type soleus is nearly zero at phase = 33.3% (Suppl. Fig. 2A), net work increased by more than 150% because positive work increased with the addition of a doublet stimulus (At phase = 33.3 %: n= 12 *mdm*, n=11 wild type trials). This result differs greatly from all other phases, making it difficult to see differences in net work between genotypes across phase. When the data from phase = 33.3% were removed (see Suppl. Fig. 2A inset), the graph shows a greater change in net work with doublet stimulation in wild type than *mdm* soleus, primarily due to the larger increase in negative work. To verify that the statistical differences between genotypes were not influenced by the trials at phase = 33.3%, we re-ran the linear mixed effects model without these data. In the absence of data at phase = 33.3 %, there was a significantly greater change in net work with doublet stimulation in wild type than *mdm* soleus ($F = 24.3$, $p < 0.0001$).

Supplementary Tables

Supplementary Table 1. Results of a linear mixed effects model on relative variables (% control) following doublet stimulation in wild-type and *mdm* soleus muscles

	**Peak Stress (% control)**		**Net Work (% control)**		**Negative Work (% control)**		**Positive Work (% control)**	
Model effects	F	*p*	F	*P*	F	*p*	F	*p*
Genotype	**23.7**	**<0.0001**	0.87	0.36	**43.48**	**<0.0001**	**9.85**	**0.004**
Phase	0.080	0.78	0.20	0.65	2.14	0.15	1.13	0.29
$Phase^2$	0.061	0.81	0.25	0.61	2.99	0.09	2.24	0.14
Genotype x Phase	2.28	0.13	**7.84**	**0.006**	**14.84**	**0.0002**	**10.17**	**0.002**
Genotype x $Phase^2$	0.49	0.48	**9.33**	**0.003**	**9.95**	**0.002**	**17.10**	**0.0001**

Bold type indicates significance; $Phase^2$ = quadratic term for phase; n = 112, df =1,29

Supplementary Figures

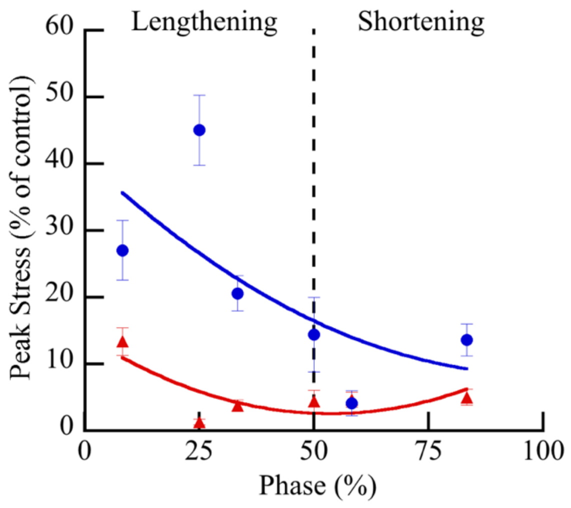

Supplementary Figure 1. **Relative increase in peak stress with vs. without doublet stimulation across phase of stimulation in wild type (blue) and *mdm* (red) soleus**. The relative increase in peak stress is reduced in *mdm* muscles compared to wild type across all phases of activation ($F = 23.7$, $p < 0.0001$). Control = trials without doublet stimulus. Phase = 0% indicates a muscle at its shortest length at the onset of lengthening and phase = 50% indicates a muscle at its longest length just prior to shortening. Data represent means ± SE, N = 29 individuals, 112 trials.

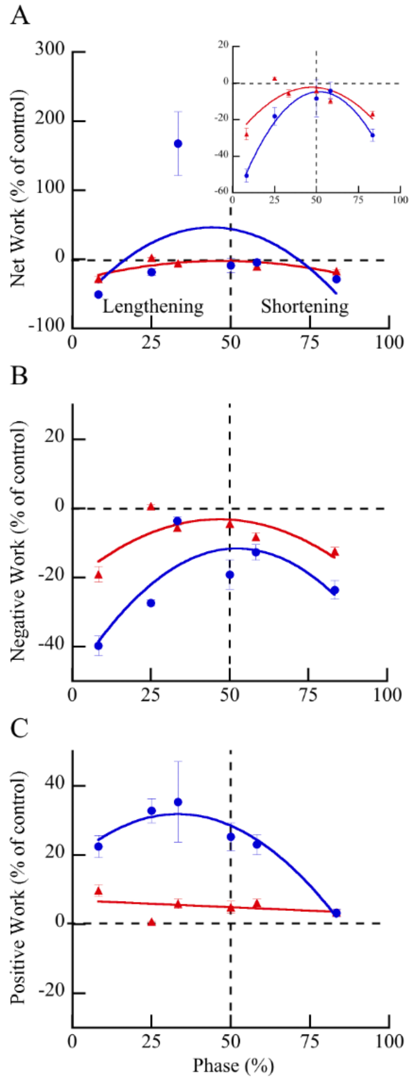

Supplementary Figure 2. **Relative differences in net (A), negative (B) and positive (C) work per cycle with vs. without doublet stimulation across phases of activation in wild type (blue) and *mdm* (red) muscles**. In general, the greatest increase in work with doublet stimulation occurred when muscles were activated during lengthening and the smallest increase occurred when muscles were activated during shortening. Wild type muscles exhibited a significantly greater relative increase in negative ($F = 43.38$, $p < 0.0001$) and positive ($F = 9.85$, $p = 0.004$) work than *mdm* muscles across all phases of activation. Because net work of wild type soleus is nearly zero at phase = 33.3% (A), net work increased by more than 150% because positive work increased with the addition of a doublet stimulus. When the data from phase = 33.3% were removed (inset), there was a greater change in net work with doublet stimulation in wild type than *mdm* soleus, primarily due to the larger increase in negative work. To verify that the statistical differences between genotypes were not influenced by the trials at phase = 33.3%, we re-ran the linear mixed effects model without these data. Even in the absence of data at phase = 33.3%, there was a significantly greater change in net work in wild type than *mdm* soleus ($F = 24.3$, $p < 0.0001$). Control = trials without doublet stimulus. Phase = 0% indicates a muscle at its shortest length at the onset of lengthening and phase = 50% indicates a muscle at its longest length just prior to shortening. Data represent means ± SE, N = 29 individuals, 112 trials.

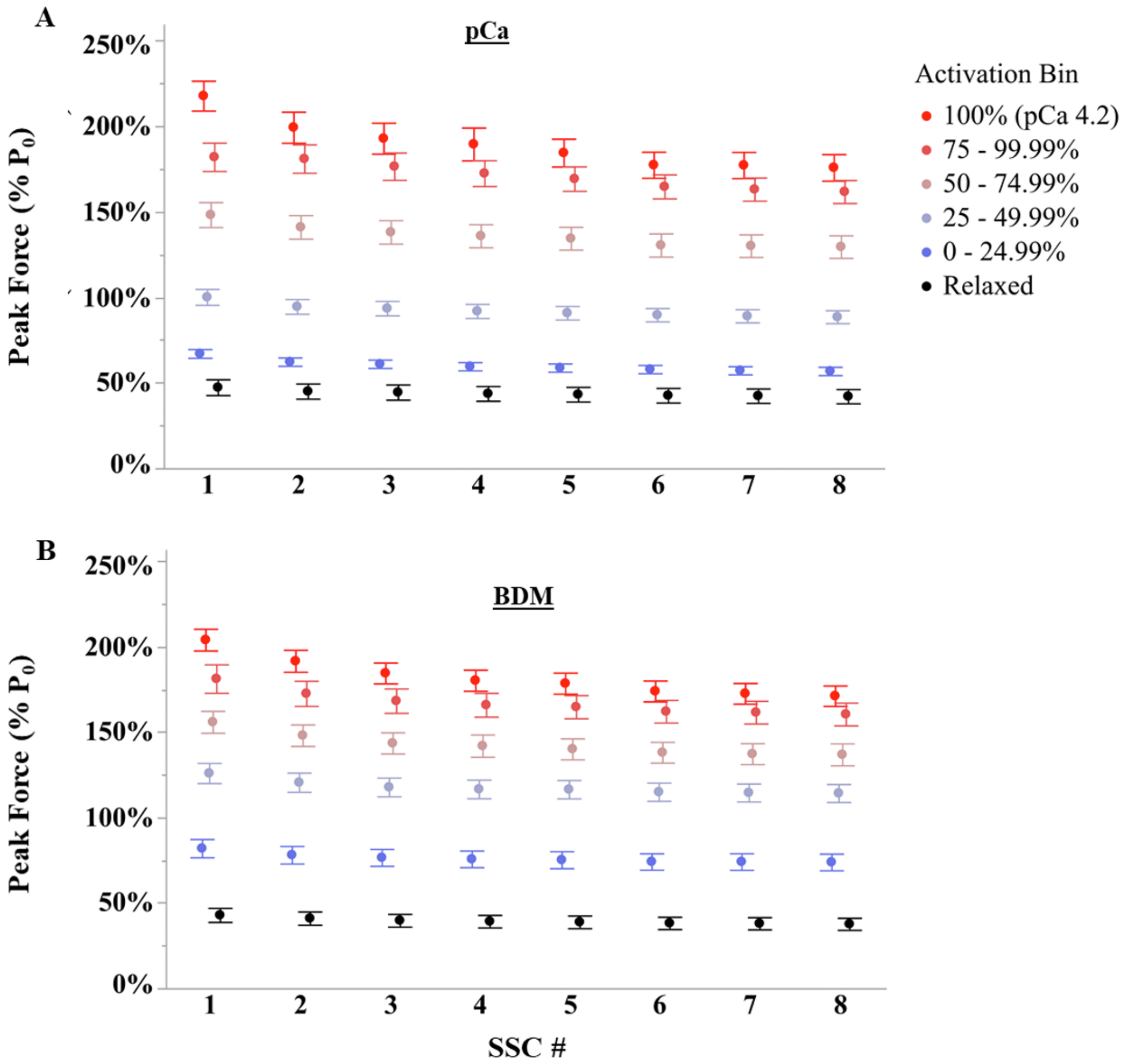

Supplementary Figure 3. Peak force by SSC # for pCa- (A) and BDM- (B) controlled fibers. Peak force is scaled relative to the isometric force at pCa 4.2 (P_0). Data were binned by activation level. Data represent means ± SEM. N = 15 pCa and 16 BDM experiments, n = 87 pCa and 100 BDM trials.
